# Association of the 2021 Child Tax Credit Advance Payments With Low Birth Weight in the US

**DOI:** 10.1001/jamanetworkopen.2023.27493

**Published:** 2023-08-09

**Authors:** Claire E. Margerison, Yasamean Zamani-Hank, Ralph Catalano, Katlyn Hettinger, Timothy R. Michling, Tim A. Bruckner

**Affiliations:** 1 Department of Epidemiology and Biostatistics, College of Human Medicine, Michigan State University, East Lansing; 2 Now with Department of Family Medicine, College of Human Medicine, Michigan State University, East Lansing; 3 School of Public Health, University of California, Berkeley; 4 Department of Economics, Michigan State University, East Lansing; 5 Program in Public Health, University of California, Irvine

## Abstract

**Importance:**

Infants and pregnant people in the US fare worse on almost all health measures compared with those in peer nations. Families in the US are more likely to live in poverty and have a less generous social safety net, which has generated debate over the contribution of economic conditions to this disparity.

**Objective:**

To assess the association between temporary increases in income during pregnancy through the 2021 expanded Child Tax Credit (CTC) and birth outcomes.

**Design, Setting, and Participants:**

This cross-sectional study applied a comparison-population, interrupted time series design to data from US birth certificates (January 1, 2014, through December 31, 2021) to test whether the log odds of low birth weight (LBW) among monthly cohorts of births exposed to the CTC would coincide with a decreased incidence of LBW. All singleton live births to US residents aged 15 to 49 years with available data were included.

**Exposure:**

Monthly birth cohorts exposed to the CTC were defined as those born to parous people during the CTC advance payment period from July through December 2021.

**Main Outcomes and Measures:**

The main outcome was the natural logarithm of the odds of LBW (<2500 g) among monthly birth cohorts.

**Results:**

Among included births (n = 28 866 466), 61.2% were to parous people, the majority were to people aged 20 to 39 years (91.7%), and 6.5% were born LBW. The odds of LBW increased above expected values in 5 of the 6 months of the CTC payments (range of increases, 3.3%-5.4% across the 5 months). The outlier-adjusted odds of LBW increased, on average, by 4.2% (95% CI, 2.7%-5.7%) among the monthly birth cohorts exposed to the CTC.

**Conclusions and Relevance:**

This study found that the odds of LBW among birth cohorts exposed to the CTC increased above expected values in 5 of the 6 months of the CTC advance payments. Additional research is needed to evaluate rival explanations for this increase in LBW among births exposed to the CTC payments.

## Key Points

**Question:**

Were the Child Tax Credit (CTC) advance payments that were disbursed in July through December 2021 associated with low birth weight (LBW)?

**Findings:**

In this cross-sectional study of more than 28 million births to parous people, the odds of LBW increased by a significant 4.2% among births exposed to the CTC (ie, monthly cohorts of births between July and December 2021).

**Meaning:**

These findings suggest that CTC advance payments did not translate to reductions in LBW, and additional research is needed to evaluate possible explanations for the associated increase in LBW.

## Teaser

This cross-sectional study assesses the association of the 2021 Child Tax Credit advance payments to parous people during pregnancy with low birth weight.

## Introduction

Compared with other high-income nations, infants and pregnant people in the US fare worse on measures of low birth weight (LBW), preterm delivery, infant mortality, and maternal mortality.^1,2^ Families in the US are also more likely to live in poverty and have a less generous social safety net, leading to debate over the contribution of economic conditions to this disparity.^3,4,5^ We contribute to this debate by testing the hypothesis that a relatively generous income transfer program in the US would coincide with a decrease in the incidence of LBW.

Income transfer programs in the US provide money to low-income families either through tax credits or basic income supplements. These programs differ in terms of eligibility requirements and amount and frequency of payments, but they are designed to support increased consumption and asset building.^6,7,8^ Prior research in the US and Canada has reported that income transfers during pregnancy increase birth weight and decrease the risk of LBW,^9,10,11,12,13,14,15,16,17,18^ albeit with some exceptions^19,20,21^ and subgroup differences.^9,21,22,23^

In response to the adverse economic effects of the COVID-19 pandemic, the American Rescue Plan Act (ARPA) expanded an important income transfer program, the Child Tax Credit (CTC).^24,25^ The ARPA increased CTC eligibility to children up to age 17 years and raised the maximum credit from $2000 to $3600 per child younger than 6 years and $3000 per child aged 6 to 17 years. Most important for our test, ARPA also changed the format of CTC payments such that instead of waiting until families had filed 2021 taxes, families who had filed taxes in 2020 or who signed up through the Internal Revenue Service’s nonfiler tool received CTC advance payments in the form of a check (ie, an income transfer) each month between July 1 and December 31, 2021. The far-reaching expansion of the CTC policy affected American families with 1 or more children and incomes up to $169 000 for those married and filing jointly, with smaller payments at higher income levels.^26^ More than 55% of American adults with children reported receiving CTC advance payments. These payments reportedly reduced the number of children living in poverty by 34%^27^ and reduced household food insufficiency by 26%.^28^

The timing and nature of the CTC expansion enable assessment of the association between temporary increases in income during pregnancy and birth outcomes. Pregnant people who already had at least 1 dependent child began receiving income transfers in July 2021, whereas those who delivered infants prior to July 2021, as well as those without a dependent child during their pregnancy, did not receive such transfers during pregnancy and, therefore, serve as comparison groups. Because changes in maternal nutrition can affect fetal growth,^29,30^ we hypothesized that the documented improvement in population nutrition associated with CTC payments^28^ could have increased fetal growth among those eligible for the CTC. We therefore assessed whether the 2021 CTC expansion coincided with a reduced incidence of LBW among monthly birth cohorts between July and December 2021 to people eligible for the CTC.

## Methods

### Data and Sample

In this cross-sectional study, we used deidentified data from the National Center for Health Statistics Vital Statistics birth certificate records from January 1, 2014, through December 31, 2021 (most recent available data), which included all live births in the US (N = 30 656 644).^31^ We included singleton births to US residents aged 15 to 49 years (n = 29 535 096). We excluded records missing length of gestation or birth weight and records with implausible combinations of birth weight and gestational age^32^ (n = 375 469), records from the unrevised (1989) version of the US birth certificate (n = 200 895), and records missing data on previous live birth (n = 92 266) for a final sample size of 28 866 466. We obtained self-reported data on maternal race and ethnicity from the birth certificate in order to describe the analytic population. This study received exempt institutional review board approval from Michigan State University with informed consent waived because the data are publicly available and deidentified. This report follows the Strengthening the Reporting of Observational Studies in Epidemiology (STROBE) reporting guideline for cross-sectional studies.

### Measures

We specified our dependent variable as the natural logarithm of the odds of LBW (<2500 g) among 96 monthly birth cohorts to parous people from January 2014 through December 2021. Available data allowed us to observe births exposed to the CTC payments during the second and third trimester of gestation (ie, births from July to December 2021). Our hypothesis assumed that the documented improvement in population nutrition associated with CTC transfers increased fetal growth among pregnant people eligible for the CTC. Low birth weight, however, reflects both fetal growth and length of gestation. We therefore focused our test on monthly birth cohort–level fetal growth by controlling for the odds of preterm birth in the same cohort.

Parents with a dependent child are eligible for the CTC. We therefore defined the exposed group as births to people with a previous live birth (as indicated on the birth certificate) since people with a previous live birth (ie, parous people) are more likely to have a dependent child compared with people without a previous live birth (ie, nulliparous people). As a comparison group, we used births to nulliparous pregnant people, because they are less likely to qualify for the CTC.

### Statistical Analysis

We used a comparison-population, interrupted time series design (an ecologic study design) to test the hypothesis that the log odds of LBW among monthly cohorts born to parous people after the CTC payment (ie, July-December 2021) would differ from their expected values. We derived expected values from preterm births among parous people during CTC payments, LBW among births to nulliparous people (the comparison group) during CTC payments, and autocorrelation in LBW among births to parous people prior to CTC payments. We further adjusted for shocks to (ie, outliers in) LBW that occurred prior to the CTC payments, such as those that could have arisen from the COVID-19 pandemic. We implemented this design using the following steps (further detail is provided in the eMethods in Supplement 1):

1. For the 90 monthly birth cohorts born before the first CTC prepayment (ie, January 2014 through June 2021), we regressed our dependent variable on the log odds of LBW to nulliparous people and on the log odds of a preterm birth (<37 weeks’ gestation) among parous people.
2. We used Box-Jenkins methods to identify autocorrelation in the residuals of the regression estimated in step 1. These methods, widely used in engineering and in the natural as well as social and health sciences,^33^ detect secular trends, cycles (eg, seasonality), and the tendency to remain elevated or depressed or to oscillate after high or low values. Any autocorrelation detected in this step logically appears only among low-weight births to parous people because the step 1 regression controls for autocorrelation shared with low-weight births to nulliparous people. We adjusted for autocorrelation because perinatal outcomes, when measured in aggregate within monthly cohorts, show well-documented seasonality, secular trends, and other patterns,^34^ and births to parous people may exhibit autocorrelation different from that in births to nulliparous people.
3. For 90 cohorts born before the first CTC payment, we estimated a Box-Jenkins transfer function formed by adding to the regression model estimated in step 1 any parameters needed to fit the autocorrelation identified in step 2. The residuals of the transfer function (ie, counterfactual values subtracted from observed values) gauge the degree to which cohorts differed from their statistically expected value based on the time series model. (Residuals also meet the assumption of a normal and independent distribution with a mean of 0. The positively and negatively signed product of 1.96 and the residual series’ SD therefore define the 95% detection interval of the residuals.)
4. With coefficients fixed to those estimated in step 3, we applied the transfer function to all 96 monthly birth cohorts, including those exposed to the CTC and born in July through December 2021. If prepayment of the CTC did not change the incidence of LBW to parous people, the residuals of the estimation would fall within the 95% detection interval estimated in step 3. We graphed the residuals as well as the 95% detection interval estimated in step 3. We then inferred that among the 6 exposed monthly birth cohorts, those, if any, with residuals outside the detection intervals yielded an unexpectedly high or low incidence of LBW.
5. We estimated the association of the CTC payment with the odds of LBW by adding a binary variable scored 1 for August through December 2021 and 0 otherwise to the step 4 transfer function and estimating the coefficients for all 96 test months.
6. We applied outlier management methods^35^ to detect and control any unexpectedly low values of low-weight births immediately preceding CTC payments.

The data management was performed using SAS, version 9.4 statistical software (SAS Institute Inc), and the time series analyses were performed using SCA Statistical System, version 5.2 (Scientific Computing Associates). A 2-sided *P* < .05 was considered significant.

## Results

**Figure 1.**
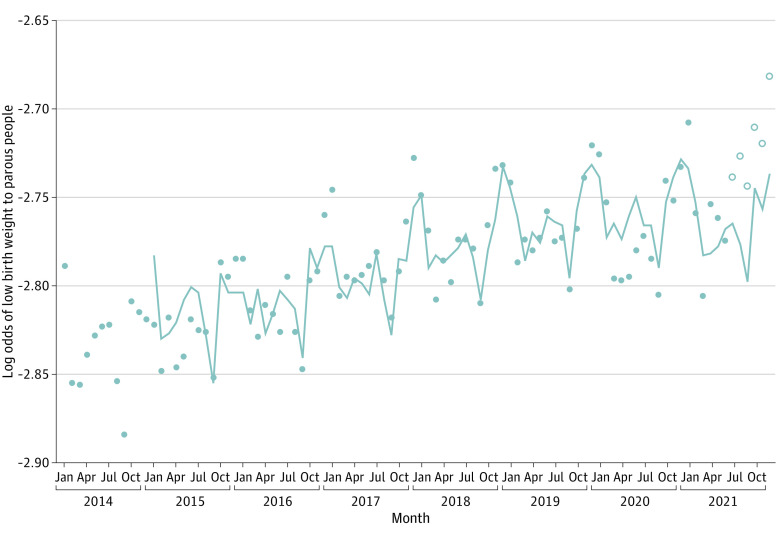
Observed and Expected Log Odds of Low Birth Weight to Parous People in the US, January 2014 through December 2021 Points represent observed values, and the line represents expected values. Open points show cohorts exposed to the Child Tax Credit benefit.

Parous people contributed 61.2% of 28 866 466 included births during the study period (Table 1). Most births (91.7%) were to people aged 20 to 39 years (vs 5.1% to those aged 15-19 years and 3.2% to those aged 40-49 years). Overall, 0.9% of births were to American Indian or Alaska Native people, 6.6% to Asian or Pacific Islander people, 13.9% to non-Hispanic Black people, 28.9% to Hispanic people, and 49.6% to non-Hispanic White people; race and ethnicity was missing or unknown for 0.3% (Table 1). The crude prevalence of LBW in the analytic population was 6.5% (Table 1). Among parous people, the prevalence of LBW was 5.8% (n = 1 024 153), which translates to a mean odds of 0.062 (range over 96 test months, 0.056-0.068). The natural log odds of LBW, our dependent variable, appear in Figure 1 as points.

**Table 1.** Descriptive Characteristics of Live, Singleton Births to US Residents Aged 15 to 49 Years With Available Data, 2014-2021

Characteristic of pregnant person	No. (%)
Parity	
Nulliparous (no previous live birth)	11 217 025 (38.9)
Parous (any previous live birth)	17 649 441 (61.2)
Age, y	
15-19	1 475 567 (5.1)
20-29	14 097 655 (48.8)
30-39	12 368 254 (42.9)
40-49	924 990 (3.2)
Race and ethnicity	
American Indian or Alaska Native	202 411 (0.9)
Asian or Pacific Islander	1 553 151 (6.6)
Black	3 285 341 (13.9)
Hispanic	6 833 977 (28.9)
White	11 737 998 (49.6)
Missing or unknown	64 175 (0.3)
Education	
Less than high school	931 922 (3.2)
Some high school	2 806 419 (9.7)
High school diploma or GED	7 341 802 (25.4)
Some college	8 138 280 (28.2)
Bachelor’s degree	5 830 988 (20.2)
More than college	3 448 038 (11.9)
Missing or unknown	369 017 (1.3)
Low birth weight	1 876 679 (6.5)

Abbreviation: GED, General Educational Development test.

The results of steps 1 through 3 of our test, in which we identified and estimated a Box-Jenkins transfer function for the log odds of LBW among the 90 pre-CTC monthly birth cohorts, are shown in Table 2. The log odds of LBW among parous people (Figure 1) and among the comparison population (ie, nulliparous people) shared a rising trend. Regressing (ie, step 1) the former on the latter, and on preterm birth among parous people, produced residual LBW values that were stationary in their mean. As shown in Table 2, we also detected (ie, step 2) seasonality peculiar to LBW among parous people and specified this autocorrelation in the transfer function.

**Table 2.** Estimated Coefficients for Low Birth Weight in US Birth Cohorts During the 90 Months Before the Disbursement of the Child Tax Credit, January 2014 Through June 2021^a^

Parameter	Coefficient (SE)	*P* value
Log odds of preterm among births to parous people	0.14 (0.07)	.053
Log odds of low birth weight among births to nulliparous people	−0.13 (0.06)	.046
Autoregression at 12 mo (ie, seasonality)	0.71 (0.07)	<.001

^a^ Estimated coefficients from a time series model of monthly log odds of low birth weight among parous people.

The results of step 4, in which we applied the step 3 transfer function to all 96 cohorts, appear as a line in Figure 1. The last 6 values of the line show the log odds of LBW that would have appeared had CTC advance payments had no association with the mechanisms that produced LBW in the previous 90 cohorts. The observed LBW values, shown as points, for the last 5 cohorts appear greater than expected (range of increases, 3.3%-5.4% across the 5 months).

**Figure 2.**
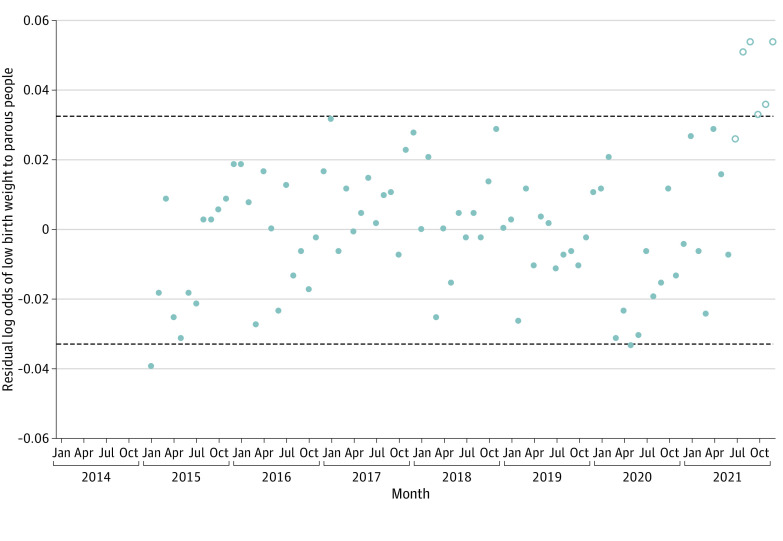
Residuals and 95% Detection Interval for Log Odds of Low Birth Weight Among Parous People in the US, January 2014 through December 2021 Open points show cohorts exposed to the Child Tax Credit benefit. Horizontal lines represent the 95% detection interval of the residuals of the transfer function, which was fitted to the log odds of low birth weight.

Figure 2 shows the residuals of LBW (ie, the difference between the observed and expected values shown in Figure 1) for each monthly birth cohort as well as their 95% detection interval. The values for the last 5 cohorts appear at or above the upper bound of the interval. We infer from this graph that more LBW infants than expected were born to parous people in the last 5 months of 2021.

The outlier-adjusted regression (ie, step 6) yielded a coefficient for the CTC binary variable of 0.042 (SE, 0.008). This estimate implies that the odds of LBW increased, on average, by 4.2% (95% CI, 2.7%-5.7%) among the cohorts born to parous people in the last 5 months of 2021. Application of the observed residual values of the odds of LBW for the last 5 months of 2021 to births to parous people in these months yielded an estimated 2295 excess LBW infants statistically associated with the CTC.

## Discussion

In this cross-sectional study, we found, contrary to our hypothesis, that the odds of LBW increased above expected values in 5 of the 6 monthly birth cohorts during the CTC advance payment distribution period among births exposed to the payment (ie, births to parous people). These findings differ from previous studies in the US and Canada that showed positive associations of income transfers with birth weight and reductions in LBW.^9,10,11,12,14,15,16,17,18,21,22,23^ Few previous studies, however, accounted for time-dependent autocorrelation in the outcome in the test group (which is particularly complex for perinatal outcomes^34^), identified a clear comparison group, or targeted the exact timing of receipt of income transfers, as we have done in this study. Moreover, other income transfer programs differ from the CTC in terms of eligibility, timing, and enrollment, and findings in Canada may differ due to the country’s more generous social safety net. Our findings are consistent with previous work showing an increased risk of very LBW in the months following the Earned Income Tax Credit (EITC) in California.^19^ The EITC shares several similarities with the structure of the 2021 CTC in its acute payout structure and focus on parous persons, although income requirements to receive the EITC are lower, so CTC recipients would have, on average, higher income than recipients of the EITC.

While the CTC payments have been shown to reduce child poverty^27^ and food insufficiency,^28^ our findings suggest that these benefits did not translate into a reduced risk of LBW. We provide several post hoc explanations for the findings. First, although the CTC was associated with decreased food insecurity, families may have used the payments to benefit existing children and not the pregnant person.^36,37^ Use of the CTC payments to benefit existing children would not, however, suggest the observed increase in LBW but would instead suggest a null result.

Second, demographic research has reported a statistically detectable spike in fertility following the COVID-19 pandemic shutdowns.^38^ Intuition suggests that this perturbation could have temporally lowered the rate of LBW if it reflected a transient shift in the composition of the reproducing population. Such a lowering could, in turn, have lowered the expectation of LBW in subsequent cohorts, including those exposed to CTC prepayments. Higher-than-expected LBW in the CTC cohorts could, therefore, arise as an artifact of the earlier pandemic rather than as an outcome of the CTC. However, in step 6 of our test, we controlled for the effect of outliers prior to the CTC payments.

Third, some literature has suggested that cash transfers are associated with increased risky health behaviors,^39,40,41^ although we are unaware of evidence in pregnant populations. However, if even a small subset of pregnant people or people in their households used the CTC in ways that negatively influenced health (eg, purchasing tobacco, alcohol, or other substances) or experienced increased stress due to receipt of the CTC, this could result in the observed increase in LBW at the aggregate level.

A fourth post hoc explanation arises from the theory of selection in utero.^42^ This theory posits that natural selection–conserved mechanisms spontaneously abort smaller fetuses (who, historically, disproportionately died before reproductive age), particularly in suboptimal or threatening environments. The standard for how large a fetus must be to avoid spontaneous abortion presumably varies over time. In more threatening environments, the standard increases, resulting in a greater proportion of the cohort being spontaneously lost but a more robust live birth cohort. In resource-rich environments, the standard drops, enabling a greater proportion of smaller fetuses to survive to live birth. The CTC benefit, therefore, could have decreased the standard, implying that small fetuses that would otherwise have been spontaneously aborted survived to birth but, in doing so, increased the odds of LBW in their monthly birth cohorts. We await the release of individual-level birth and fetal death files in coming years to further assess this and other post hoc explanations and to examine birth outcomes after the expanded CTC payments ended in January 2022.

### Strengths and Limitations

Strengths of the analysis include our ability to adjust for seasonality specific to parous people who are pregnant and to control for any temporal patterns in LBW that occur generally across both parous and nulliparous people. By controlling for odds of preterm birth in the exposed group, we also reduce the potential that unexpected values of LBW are driven by increased preterm birth in the cohort. Furthermore, the acute timing of the CTC minimizes the possibility that selection into pregnancy accounts for the results.

The study also had several limitations. We lacked data on the cash value of CTC received; thus, our identification strategy is like an intention-to-treat framework used for clinical trials.^43^ The individual-level association of the CTC payment with LBW among those who received the credit may differ from the average outcome in the exposed population (parous pregnant people). We also could not ascertain trimester of exposure using gestational age dating, which implies that births in our exposed cohorts may have had varying exposure time to the CTC. We await data on births occurring in 2022 (exposed to the CTC payments in the first trimester) to further explore timing of payment during pregnancy.

Any test based on other than random assignment has the inherent limitation that some uncontrolled confounder could spuriously induce results. To evade control in our test, however, a confounder would have to affect LBW only among parous (compared with nulliparous) people, have no effect on preterm birth, exhibit no autocorrelation (including seasonality), and appear coincidentally with CTC prepayments. We know of no confounder that meets these criteria.

## Conclusions

The findings of this study suggest that the relatively generous, but temporary, advance CTC payments received by more than one-half of US families between July and December 2021 corresponded with an increase in odds of LBW. Our findings suggest that the documented reduction in poverty associated with the CTC did not translate to reduced LBW. The theory of selection in utero would support that the CTC could have reduced the number of infants spontaneously lost prior to birth, resulting in a greater proportion of small infants surviving to live birth. We encourage replication and extension of our work to examine the outcomes of the CTC expansion on parental and child health domains over the life course, which may be complex and varied based on prior examinations of income transfers on health.^8^ Such work could help to inform ongoing policy considerations, such as a permanent child allowance or universal basic income programs.

## Notes

Supplement 1.
eMethods.

eReferences

Supplement 2.
Data Sharing Statement
