# Supplementary material for: Association of the 2021 Child Tax Credit Advance Payments With Low Birth Weight in the US
Source: JAMA Netw Open. 2023 Aug 9;6(8):e2327493. doi: 10.1001/jamanetworkopen.2023.27493 (PMC10413172; doi:10.1001/jamanetworkopen.2023.27493)
Supplement: Supplement 1. — eMethods. eReferences [file jamanetwopen-e2327493-s001.pdf]

Supplemental Online Content

Margerison CE, Zamani-Hank Y, Catalano R, Hettinger K, Michling TR, Bruckner TA. Association of the 2021 Child Tax Credit advance payments with low birth weight in the US. *JAMA Netw Open*. 2023;6(8):e2327493. doi:10.1001/jamanetworkopen.2023.27493

eMethods.

eReferences

This supplemental material has been provided by the authors to give readers additional information about their work.

© 2023 Margerison CE et al. *JAMA Network Open.*

eMethods.

Step 1. *What we did* - For the 90 monthly birth cohorts born *before* the first CTC prepayment (i.e. January, 2014 through June, 2021), we regressed the log odds of low birth weight (LBW) to parous people on the log-odds of a preterm birth (<37 weeks gestation) among parous people and the log-odds of LBW to nulliparous people.

Why we did this – We did this because estimating the pre-CTC associations among these 3 variables allows for subsequent (i.e., Step 4) control of two potentially confounding sources of temporal variation in the log odds of LBW in monthly cohorts born to parous people. First, by specifying nulliparous people as our comparison population, it allows control of "third variables" that influence monthly odds of LBW among all pregnant people regardless of parity. These variables can induce not only trends and cycles (e.g., seasonality), but also "sporadic" high or low values that appear for a single birth cohort or that persist, although diminished, into subsequent cohorts. Changes in these third variables could have coincided with the CTC thereby inducing a spurious association. Second, the Step 1 regression allows control (in Step 4) for the frequency of preterm birth in monthly birth cohorts. Preterm birth reduces birthweight and the frequency of preterm birth among births to parous people could have coincided with the CTC thereby inducing a spurious association with LBW.

Results – the regression equation produced the following estimated coefficients for the 90 pre-CTC cohorts

$$Y_t = 1.146 + 0.447X_{1t} + 0.239X_{2t} + e_t \quad \text{[Eq 1]}$$

In which Yt is the log odds of LBW to parous people in month t. 1.146 is a constant. X_{1t} is the log odds of LBWto nulliparous people in month t. X_{2t} is the log odds of preterm birth to parous people in month t. e_t is the residual at month t.

Step 2. *What we did* – We used Box-Jenkins methods to identify autocorrelation in the residuals of the regression estimated in Step 1. These methods, widely used in engineering and in the natural as well social and health sciences[1], detect secular trends, cycles (e.g., seasonality), and the tendency to remain elevated or depressed, or to oscillate, after high or low values. Any autocorrelation detected in this step logically appears only among low-weight births to parous people because the Step 1 regression controls for autocorrelation shared with low-weight births to nulliparous people.

Why we did this – Autocorrelation specific to LBW to parous people could have yielded a high or low value that coincided the CTC and thereby induced a spurious association. Step 2 controls for autocorrelation in LBW among parous people only.

Results – Box Jenkins methods detected seasonality in that the log odds of LBW among parous births in month t predicted the log odds at month t+12.

© 2023 Margerison CE et al. *JAMA Network Open.*

Step 3. *What we did* -- For 90 cohorts born before the first CTC distribution, we estimated a Box-Jenkins "transfer function" formed by expanding the regression equation estimated in Step 1 to include an autoregressive parameter at t-12 needed to fit seasonality identified in Step 2.

Why we did this – We did this to generate a time-series model that estimates the expected log odds of LBW, in monthly cohorts born to parous people, based on the frequency of preterm birth in the cohort, phenomena that affect the likelihood of LBW among pregnant people regardless of parity, and seasonality in LBW to parous persons. The residuals of this model, which exhibit no autocorrelation (i.e., are independent of each other), are normally distributed, and have a mean of 0, gauge the degree to which the observed values in any cohort differed from expected. The positively and negatively signed product of 1.96 and the residual series' standard deviation therefore define the 95% detection interval of the residuals.

Results – The estimated parameters of the transfer function were as follows:

$$(1-0.711B^{12})[Y_t-(-2.791)] = -0.128X_{1t}+0.141X_{2t}+e_t \quad \text{[Eq 2]}$$

In which 0.711 is the autoregressive coefficient. B^{12} is the seasonal "backshift operator" indicating that the autoregressive coefficient applies the value of Y at t-12. -2.79 is the mean of Y for 90 cohorts. The other parameters are defined identically to those specified in Eq 1. (i.e., X_{1t} is the log odds of LBW to nulliparous people in month t. X_{2t} is the log odds of preterm birth to parous people in month t. e_t is the residual at month t.)

Step 4. *What we did* -- With coefficients fixed to those shown in equation 2 above, we applied the transfer function *to all 96 monthly birth cohorts including those exposed to the CTC* and born in July through December.

Why we did this – We did this because the last 6 fitted values of the transfer function serve as counterfactuals, or expected values of monthly log-odds of LBW, had the CTC not occurred. The last 6 *residuals*, therefore, gauge the degree to which cohorts exposed to the CTC differed from expected.

By axiom, the first 90 residuals of this estimation would be identical to those of the Step 3 estimation. As noted above, they would have a mean of 0 and be independent of each other. If the CTC did not change the circumstances that determine LBW to parous people in the United States, the last 6 of the 96 residuals of the Step 4 estimation would fall within the interval defined by the positively and negatively signed product of 1.96 and the standard deviation of the first 90 residuals (i.e., the 95% detection interval).

Results – Figure 2 in the manuscript shows the last 6 residuals and the 95% detection interval. As shown, 5 of the last 6 residuals appear above the upper bound of the detection interval.

Step 5. *What we did* -- We quantified the association between CTC payment and odds of LBW by adding a binary variable scored 1 for August through December 2021 and 0 otherwise to the

© 2023 Margerison CE et al. *JAMA Network Open.*

transfer function shown as equation 2 above and estimating the coefficients for all 96 test months.

> *Why we did this* – This estimation allowed us to express our results as the percentage increase in the odds of LBW associated with the CTC.
>
> *Results* – The estimated parameters were as follows.
>
> $$(1\text{-}0.709B^{12})[Y_t\text{-}(\text{-}2.787)] = \text{-}0.131X_{1t}+0.145X_{2t}+0.047X_{3t}+e_t \quad [3]$$
>
> In which X_{3t} is a binary variable scored 1 for August through December 2021 and 0 otherwise. The coefficient for the CTC binary variable (i.e., 0.047) has a standard error of 0.008. This estimate implies that the odds of LBW increased, on average, by 4.7% (95% confidence interval: 3.1, 6.3) among the cohorts born to parous people in the last 5 months of 2021 (range of increases: 3.3 to 5.4% across the 5 months).

Step 6. *What we did* -- We applied outlier management methods[2] to detect and control any unexpectedly low values of low weight births immediately ***preceding*** CTC payments.

Why we did this – The epidemiologic literature reports that preterm births in the US unexpectedly fell among cohorts born early in the pandemic. As reported in our manuscript, a similar fall appears in LBW. This fall raises the question of whether low outlying values immediately before CTC payments could have created an atypical low expectation for low weight births in our test period and thereby induced our results. Although we control for preterm births in our transfer function, we went further and searched for, and controlled, outliers in the residuals of the Step 6 estimation (i.e., equation 3 above).

Results – We found a sequence of 4 cohorts beginning April 2020 that showed unexpectedly few ($p < .01$) low weight births to parous women. Controlling for these outliers did not, however, affect our results. The outlier-adjusted parameters of equation 3 were as follows.

$$(1\text{-}0.709B^{12})[Y_t\text{-}(\text{-}2.787)] = \text{-}0.130X_{1t}+0.143X_{2t}+0.042X_{3t}+e_t \quad [4]$$

The coefficient for the CTC binary variable (i.e., 0.042) has a standard error of 0.0076. This estimate implies that the odds of LBW increased, on average, by 4.2%. Application of the observed residual values of the odds of LBW for the last 5 months of 2021 to the parous births in these months yields an estimated 2,295 excess LBW infants statistically associated with the CTC.

This estimate cannot be logically attributed to any confounder that might arise from shocks to LBW early in the pandemic, or that affects pregnant people regardless of parity, or that affects only parous people and exhibits autocorrelation (including secular trends and seasonality). Any confounding shock to gestation that survives these exclusion criteria would, moreover, induce our findings only if it coincided, by chance, with CTC payments. We know of no potential confounder that fit all these criteria.

© 2023 Margerison CE et al. *JAMA Network Open.*

eReferences

1. Helfenstein U. The use of transfer function models, intervention analysis and related time series methods in epidemiology. *Int J Epidemiol*. 1991;20(3):808-815. doi:10.1093/ije/20.3.808

2. Chang I, Tiao GC, Chung C. Estimation of Time Series Parameters in the Presence of Outliers. 1988;30(2):193-204.

© 2023 Margerison CE et al. *JAMA Network Open.*
