# Supplementary material for: Association of the 2021 Child Tax Credit Advance Payments With Low Birth Weight in the US
Source: JAMA Netw Open. 2023 Aug 9;6(8):e2327493. doi: 10.1001/jamanetworkopen.2023.27493 (PMC10413172; doi:10.1001/jamanetworkopen.2023.27493)
Supplement: Supplement 2. — Data Sharing Statement [file jamanetwopen-e2327493-s002.pdf]

Data Sharing Statement

Margerison. Association of the 2021 Child Tax Credit Advance Payments With Low Birth Weight in the US. *JAMA Netw Open*. Published August 09, 2023. doi:10.1001/jamanetworkopen.2023.27493

Data
Data available: No

Additional Information
Explanation for why data not available: These data are already publicly available from the National Center for Health Statistics.
